# The Assessment of Knowledge about Tourette's Syndrome among Medical Students and Primary Physicians in Riyadh, Saudi Arabia: A Cross-Sectional Study

**DOI:** 10.1155/2022/3018305

**Published:** 2022-02-28

**Authors:** Anas A. Alalwan, Mohammad A. Alkhamis, Ahmad M. Samman, Enan H. M. Alsharif, Omar E. M. Tarabzoni, Ismail A. Khatri

**Affiliations:** ^1^College of Medicine, King Saud Bin Abdulaziz University for Health Sciences, Riyadh, Saudi Arabia; ^2^Division of Neurology, Department of Medicine, King Abdulaziz Medical City, MNGHA, Riyadh, Saudi Arabia; ^3^King Abdullah International Medical Research Center (KAIMRC), Riyadh, Saudi Arabia

## Abstract

**Background:**

Tourette's syndrome (TS), a chronic, often disabling neuropsychiatric disorder characterized by motor and vocal tics, is frequently misdiagnosed, or patients are delayed in diagnosis. There is severe deficiency of research about Tourette's syndrome (TS) in the Middle East region.

**Objectives:**

To evaluate the knowledge and attitude of medical students and primary care physicians (PCPs) about TS and tic disorders.

**Methods:**

IRB approved, cross-sectional study. A total of 316 medical students of King Saud bin Abdulaziz University and 59 primary care physicians of Riyadh participated. Convenient, cluster sampling was used. A validated, self-administered questionnaire was used. Sum of all knowledge questions was calculated. Data were analyzed using SPSS software.

**Results:**

Survey was completed by 375 students and physicians, of whom 253 (67.5%) were men. Mean general knowledge score was 61.5 (±12.04) out of 100. Majority (66.1%) knew the diagnostic criteria for TS; only 46.1% considered antipsychotics as effective treatment. Only 25.1% had ever heard of habit reversal; 70% wanted to learn more. Only 10% of physicians had treated a patient with TS. There was no difference in knowledge between men and women (*p*=0.776). Board-certified physicians had a higher knowledge score (*p* < 0.05). Family physicians demonstrated higher level of knowledge compared to other physicians (*p* < 0.05). There was no difference between knowledge of students of different years (*p*=0.859) or between students and physicians (*p*=0.569).

**Conclusion:**

There was alarming lack of knowledge about Tourette syndrome at various level of medical training and practice including students and physicians. Those who achieved board certification and practiced as family physicians fared better in knowledge about Tourette's syndrome.

## 1. Introduction

Tourette's syndrome (TS) is a chronic, neuropsychiatric disorder, in which the patients develop multiple motor and vocal tics that last for at least once year and the onset is before the age of 18 years [1]. The typical age of onset of symptoms is between 4 and 6 years, although the most severe tics may appear later [1]. This condition was once believed to be a rare neurological disorder; however, recent studies have reported prevalence figures between 0.4% and 3.8% for the ages between 5 and 18 [2]. The overall prevalence data are debated, but the closest estimates suggest a prevalence of 0.5% (1 in 200) in children of school age; whereas, in adults, it is estimated at 0.012% (118 cases per 1 million population) [1, 3]. The diagnosis criteria for TS include multiple motor tics and one or more vocal tics that last more than a year, and the disturbances must cause distress or social difficulties [4]. The general term “tic” is defined as “a rapid, repeated, nonrhythmic movement or sound production that occurs suddenly and serves no purpose” [5]. Vocal and motor tics can be either simple or complex and can range from mild to severe, depending on factors such as the number of muscles associated with the tic and whether the tic appears coordinated [6]. People who experience tics feel a “premonitory urge,” which is a feeling of the necessity to perform specific movement(s). Although tics can be suppressed, a rebound reaction can be expected, and sometimes, it can even be stronger than the initial urge of the tic.

The majority of patients suffering from TS have a comorbidity. The most common of those comorbidities are attention-deficit/hyperactivity disorders (ADHD), learning disabilities, or obsessive-compulsive disorders (OCD) [7]. The exact etiology of TS is yet unknown, but tics are believed to have complex pathophysiology involving presynaptic and postsynaptic neurons, as well as glial cells in the central nervous system [8]. Tourette's syndrome can be managed by pharmacotherapy and nonpharmacological interventions. For tics reduction, positive behavioral programs appear to be the most beneficial, while the goal of pharmacotherapy is to enhance the patient's functioning to an acceptable level with the lowest possible dose of medication [9, 10].

Tourette' syndrome patients usually suffer from delayed diagnosis and misdiagnosis. This could be caused from the lack of awareness of the onset symptoms and the wrong beliefs that some physicians hold about TS. In many cases, TS is only diagnosed if the patient has coprolalia, which is a repetitive use of obscene language [11]. In general, coprolalia is observed in only 10–20% of TS patients [2, 12]. TS patients often experience social isolation, bullying, and discrimination caused by misperceptions of the disorder by the community [11].

There is a severe lack of research about TS in the Middle East region. Most of the studies are done in Europe, North America, and Far East. A meta-analysis and systematic review of 27 studies on TS found that only 2 studies were done in Middle East, one in 1992 and one in 2012. All the other reported studies were from Europe, North America, or Far East [13]. TS is still believed to be a rare and an obscure syndrome, despite all the evidence that points otherwise. The aim of this study was to determine the knowledge of Tourette's syndrome among medical students at King Saud bin Abdulaziz University for Health Sciences (KSAU-HS) and Primary Care Physicians (PCPs) in Primary Health Care Centers (PHCCs) that are government funded and follow the Ministry of Health (MOH). This study also aimed to determine whether medical colleges provided adequate education about TS and tic disorders in general. The findings of this study will help the medical education planners to reconsider the curriculum to include education about relatively uncommon but clinically relevant and disabling neuropsychiatric conditions.

## 2. Methods

### 2.1. Study Design and Settings

This was a cross-sectional study approved by Institutional Review Board (IRB) of King Abdullah International Medical Research Center (KAIMRC) and King Saud bin Abdulaziz University for Health Sciences (KSAU-HS). The study was conducted in the city of Riyadh, Kingdom of Saudi Arabia. The study sites were King Saud bin Abdulaziz University for Health Sciences, Riyadh, and primary health care centers (PHCCs) in Riyadh. The data were collected between June 2019 and September 2019.

### 2.2. Study Participants and Sample Size

The study participants were divided into two groups: group A and group B. Group A included PCPs. There are approximately 300 physicians distributed around 88 PHCCs in Riyadh city. Cluster sampling was used, dividing Riyadh into four sections. 50 PHCC were randomly selected, and all physicians in those centers were asked to participate. Group B included medical students of KSAU-HS, which has approximately 1100 medical students of both genders, distributed into 4 batches (year 1, year 2, year 3, and year 4). Students could be either from stream 1 or stream 2. Stream 2 students are holders of previous bachelor's degrees in scientific fields (science, applied medical sciences, and pharmacy), while stream 1 students do not have a previous degree. The reason to select medical students and PCPs for this study was that most of the medical students after graduation take the role of PCPs; hence, we thought this was an appropriate assessment of evolution of knowledge from training to practice level. The convenient sampling method was used to distribute the questionnaire among the students. Students who were in the preparatory and premedical phases and in any other specialty other than medicine, primary care, dentistry, and who refused to participate were all excluded from this study. The margin of error was chosen to be 5%, with confidence level of 95%. The sample size was calculated using Raosoft. The calculated sample size for medical students was 289 and for PCPs was 169. A total of 316 medical students and 59 PCPs completed the questionnaire. The disproportion between the number of students and number of PCPs was partly based on the sample size calculation. Out of the calculated number of PCPs, we could not obtain response from adequate number; hence, the study is somewhat underpowered.

### 2.3. Data Collection and Study Tool

Informed consent was obtained from all participants. The participants were asked to complete an acquired, previously validated, self-administered questionnaire [14]. The questionnaire was focused on assessing multiple areas of information regarding TS. First, a few general knowledge questions were asked about TS. Second, the questionnaire asked PCPs about their beliefs regarding environmental factors and untested claims and their effect on TS. Third part of the questionnaire inquired about the management, either pharmacological or nonpharmacological, and the knowledge of support groups. Fourth part comprised of assessing the familiarity with habit reversal therapy and the participants' interest in knowing more. Subsequently, a scoring system was used to quantify the amount of knowledge and information.

### 2.4. Data Analysis

The questionnaires were coded, and the data collected were entered into Excel 2020 files. Continuous data were presented as means and standard deviations. Categorical data were presented as frequencies and percentages. The *t*-test and ANOVA tests were used for statistical analysis. Any *p* value less than 0.05 was considered significant. The sum of all knowledge questions was calculated for each participant. All data were analyzed using SPSS v26 software.

## 3. Results

The total number of participants was 375 which included both physicians (*n* = 59 (15.7%)) and students (*n* = 316 (84.3%)). Out of 375 participants, 253 (67.5%) were men, and the majority were Saudis (*n* = 333 (88.8%)). The general demographics of the surveyed population and their respective knowledge scores are given in Table 1. The distribution of medical students in various years is given in Table 2 with their knowledge scores. The qualification of various physicians is given in Table 3 with their knowledge scores.

**Figure 1:**
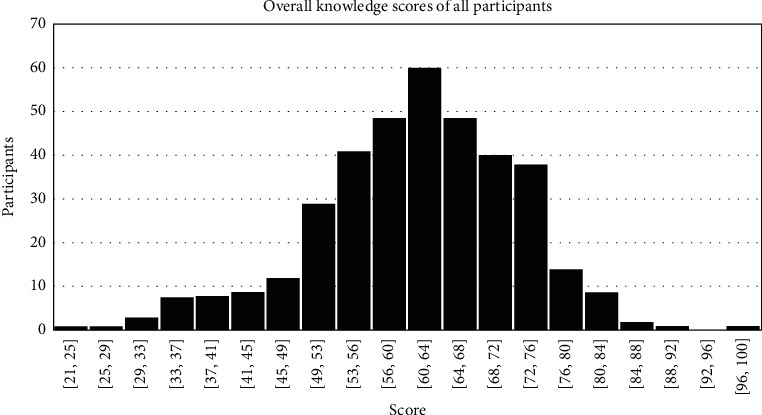
Overall knowledge scores of all participants shown in histogram.

**Figure 2:**
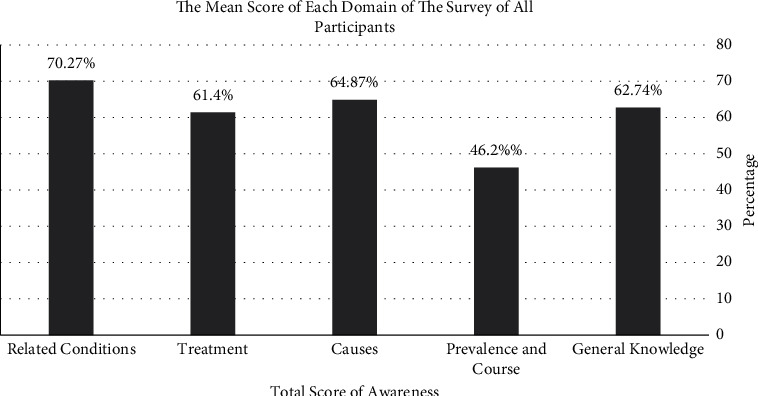
The average knowledge scores about various aspects of Tourette's syndrome.

Overall knowledge scores of the entire survey participants are shown in Figure 1. No significant difference was found in the knowledge of students from different years of training (*p*=0.859). The physicians who had postgraduate board certification had significantly better knowledge (*p*=0.003) compared with physicians without board certification, as given in Table 3. Family physicians demonstrated a higher level of knowledge compared to other physicians (*p*=0.002). The physicians who spent 5–10 years in their practice had the highest mean score among various groups of physicians; however, this did not reach a statistical significance (*p*=0.102), as given in Table 4. Majority of PCP (89.9%) had never treated a patient with TS.  Figure 2 shows the comparison of the knowledge among medical students and PCPs.

**Figure 3:**
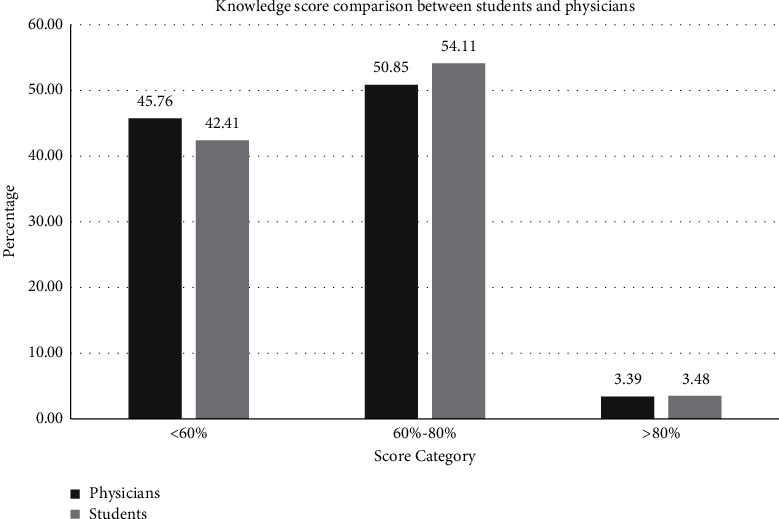
Knowledge score category comparison between students and primary care physicians.

The degree of awareness about various aspects of TS among students and physicians is shown in Figure 3. Most of participants (66.1%) knew the correct definition for tics, and most participants (66.1%) were knowledgeable about the diagnostic criteria for TS. Majority of the participants (52.8%) identified that coprolalia is present in a minority of cases of TS. The majority (68.8%) knew that TS was more common in males, and most of the responders agreed that tics are most severe during childhood (68.3%). Majority of the participants (62.7%) agreed that TS is mainly a biological disorder rather than a psychological disorder. Around half of the responders (47.5%) believed that TS is genetically transmitted. Regarding the medications used in the treatment, the statement that antipsychotics can help control the tics associated with TS was agreed by most of the participants (72.5%). Furthermore, anticonvulsants were the most selected choice (46.1%) as the most effective medication for TS, and antipsychotics were second (45.1%). More than half (52%) thought that tics worsened if they were discussed with TS patients. Most of the responders (74.1%) had not heard about habit reversal therapy (HRT) for TS. Most of the participants (85.3%) did not know how to implement the HRT, but 69.9% were interested to know about HRT. Responding to the relationship between TS and attention-deficit/hyperactivity disorder (ADHD), 64.8% of participants agreed that TS patients are more likely to have ADHD compared to general population; moreover, 55.5% agreed that TS patients have a higher risk to develop obsessive-compulsive disorder (OCD) compared to normal individuals. A national support group for TS patients was heavily advocated by nearly all responders (89.6%).

## 4. Discussion

Tourette's syndrome is underrecognized and undertreated. Our study also showed suboptimal awareness about TS among medical students and PCPs of Riyadh region. This is the first study of this kind from the region. The results showed that medical students had general knowledge about TS that was equal to that of physicians. Both physicians and students had an overall knowledge score of 61%, which is much lower than the 77% score reported by Marcks et al. [14]. This suboptimal knowledge score necessitates better education and training on how to recognize and deal with TS and tic disorders in general.

Physicians, in general, lacked the ability to accurately diagnose and manage TS. Only 10% of physicians had ever treated a patient with TS, compared with 46% in the study of Marcks et al. [14]. One of the reasons for such a low number of patients could be the inability of physicians to diagnose TS. Overall, the family physicians had higher level of knowledge about TS compared to other physicians. Although we did not study it systematically, it is possible that during family medicine residency training, there is additional emphasis on such conditions [15]. There was also a significant difference between the various certifications that the physicians had. Physicians who had board certification had higher knowledge score than physicians who did not, which probably can be explained by wider knowledge base of board-certified physicians. Only 40% of both physicians and students knew that the international prevalence of TS was around 1%, while 32% believed that it was less than 1% [16]. This further supports the idea that the majority still see TS as a rare disorder that is not relevant in the Middle East.

There was no difference between knowledge of students in different years of training. Although not statistically significant, female gender among both physicians and students demonstrated better knowledge of Tourette's syndrome. Among the respondents, 46% believed that anticonvulsants are the better medical treatment, which showed lack of understanding about managing TS. Most respondents had not heard of habit reversal therapy for TS, but both students and physicians showed high interest in learning more about it [17]. A national support group for TS patients was highly advocated by all responders.

This study has several limitations. This study was limited to one medical university; hence, the findings cannot be applied to all universities in the region. It may be worthwhile in future to study the knowledge of students of multiple universities in the region and compare that to university students in the West. We saw a low response rate from physicians which might have caused nonresponse bias and could potentially limit the generalizability and validity of the findings. One of the reasons for low response rate from physicians could be time restraints in their busy practice [18]. Increasing the number of data collectors and involving more PHCCs could increase the number of participants. Considering this study was conducted primarily on PCPs, the majority of participants were either general or family physicians. It would have been interesting to compare the different knowledge scores between different medical and surgical specialties, had those been targeted and included. The usage of convenient sampling to collect data from students might have also caused sampling bias. The large number of students and small number of data collectors could be addressed possibly by an online or virtual data collection. The fact that our university is sex-segregated made it difficult to obtain data from the female section.

## 5. Conclusions

In conclusion, the results of this study showed that the awareness about TS both among medical students and primary care physicians was alarmingly low. Board certification and career role as family physicians were associated with better knowledge. Advancement in medical school did not affect knowledge about TS. Additional education about diagnosing and managing TS could benefit both physicians and students and perhaps patients as well. More learning time dedicated to tic disorders that could help abridge the gap of knowledge should be on the agenda of medical education planners. Most importantly, more research studies about TS are needed in the Middle East, including prevalence studies.

## Figures and Tables

**Table 1:** Mean knowledge scores according to demographic variables.

	*N* (%)	Mean (±SD)	*P* value
Gender			
Male	253 (67.5)	60.77 (±12.02)	0.776
Female	122 (32.5)	63.15 (±12.00)	
Nationality			
Saudi	333 (88.8)	61.59 (±11.87)	0.723
Non-Saudi	42 (11.2)	61.21 (±13.51)	
Level of training			
Physicians	59 (15.7)	60.31 (±13.30)	0.559
Students	316 (84.3)	61.77 (±11.81)	

**Table 2:** Distribution of students in various levels of training and their knowledge scores.

	*N* (%)	Mean (±SD)	*P* value
Stream			
Stream 1	285 (90.2)	61.81 (±11.90)	0.653
Stream 2	31 (9.8)	61.49 (±11.66)	
Batch			
Batch 12 (year 4)	82 (25.9)	61.64 (±11.35)	0.859
Batch 13 (year 3)	85 (26.9)	61.37 (±10.88)	
Batch 14 (year 2)	78 (24.7)	62.77 (±12.24)	
Batch 15 (year 1)	71 (22.5)	61.33 (±13.03)	

^*∗*^Stream 2 students are holders of bachelor's degrees in scientific fields (science, applied medical sciences, and pharmacy).

**Table 3:** Knowledge scores based on the highest qualification and specialty of physicians.

	*N* (%)	Mean (±SD)	*P* value
Highest educational degree			
MBBS	30 (50.9)	58.06 (±12.18)	0.003
MD	6 (10.2)	56.25 (±10.79)	
BC	11 (18.6)	67.8 (±5.92)	
SBC	2 (3.4)	87.5 (±17.68)	
Not selected	10 (16.9)	55.83 (±15.49)	
Specialty of practice			
General practice	38 (64.4)	59.54 (±11.94)	0.002
Family physician	12 (20.3)	70.83 (±11.38)	
Pediatrician	2 (3.4)	43.75 (±26.52)	
Others	7 (11.9)	51.19 (±7.87)	

MBBS, Bachelor of Medicine and Bachelor of Surgery; MD, Doctor of Medicine; BC, board certified; SBC, Saudi board certified.

**Table 4:** Knowledge scores based on experience and prior treatment of Tourette's syndrome and tic disorders.

	*N* (%)	Mean (±SD)	*P* value
Years in practice			
<5 years	16 (27.1)	59.12 (±12.10)	0.102
5–10 years	17 (28.8)	66.67 (±11.41)	
11–20 years	17 (28.8)	58.09 (±15.41)	
>20 years	9 (15.3)	54.63 (±11.68)	
Prior treatment of patient with Tourette's syndrome (TS)			
Prior experience present	53 (89.8)	60.61 (±13.11)	0.516
Prior experience absent	6 (10.2)	57.64 (±15.9)	
Prior experience of treating patients with tic disorders other than TS			
0 patients treated	45 (76.8)	61.02 (±13.09)	0.575
1–5 patients treated	11 (18.7)	59.47 (±14.68)	
>5 patients treated	3 (5)	52.78 (±13.39)	

## Data Availability

The data used to support the findings of this study are available from the corresponding author upon request.
